# The Grass Is Always Greener, or Is It?

**DOI:** 10.1093/asjof/ojaa022

**Published:** 2020-06-04

**Authors:** Alexandra Hart

**Affiliations:** Division of Plastic and Reconstructive Surgery, Emory University School of Medicine, Atlanta, GA

Since Match Day, my fourth year of medical school, I have been thinking about the summer of 2020. During these moments, I would be basking in the final months before graduating from residency as a plastic surgeon. In my mind, I would have the perfect contract signed, the perfect international vacation planned, it would be the perfect time to graduate because of a booming economy, and I would be planning to start my job with, of course, the perfect mix of patients and problems.

Fast forward to spring of 2020, and while I am 2 months from graduating, none of those things are in the books. In fact, as I write this, I am at home trying to work as my baby is screaming in the background. I think it’s safe to say that for everyone, not just the graduating chief residents, the world today isn’t what we expected. Everyone is instructed to stay 6 feet apart, restaurants closed, businesses hurting, unemployment skyrocketing^1^…and all this because of a virus that is not only damaging the economy but also the lives of millions.

While my musings as a medical student were immature and not likely to be true even in the best of circumstances, if I reflect for a moment on what “should” have been (maybe even my expectations) as recently as February, there are still distinctions. I should be on cruise control, sailing through my last rotation, which was outpatient aesthetic surgery rotation, operating on healthy COVID-free patients. In that reflection, the grass seems greener, much greener.

As I am sure the majority of us can attest, I have answered more questions and emails in the last 2 weeks than the previous 10 months. Graduation is likely canceled. All of our lectures are through Zoom, and I am missing my elective and final aesthetics rotation. This is reality. As a resident, our job alternates between performing service-based tasks (notes, consults, call, etc.) and true educational experiences. In the world of COVID, even doing duties that are usually mundane (ie, rounding, taking a history) feel like pure service as all are accompanied with a fear of bringing this virus home to our families. As administrative chief resident, I have redone not only the call schedule but also the entire schedule…again. Like many programs, the residents are divided into 2 groups. One group is covering everything while the other stays quarantined. What does this change mean for the education of our residents?

At my program, during the last half of the year, the graduating chiefs take a step back and the upcoming class becomes the functioning chiefs at both our trauma hospital and the busy reconstructive service. Due to COVID, this is an opportunity this class of residents is missing. It’s their chance to finally pick their cases, run a service, and manage the schedule. Having had this experience myself, this is one of the greatest educational experiences offered to a resident. I know these residents are feeling anxious about this missed opportunity. Furthermore, what does this mean for the next academic year’s rotation schedule? Will this alter how the schedule is constructed moving forward taking away this opportunity for the class the following year? I can only say I hope not. Additionally, I have no doubt that my coresidents and all those around the country who feel that they are missing pivotal operative moments will be able to adapt and catch up. These “missed” experiences don’t have to be wasted time. There are other ways to learn, improve, and advance outside of the hospital.

If instead of wondering what “should” have been, we take a moment to focus on the things we have. What *is* the reality now? I have extra time, something I haven’t had in over 10 years. Time to sit and drink coffee with my husband. Time to read my baby a book… or 6. Time to finish the research projects that have been on my to-do list for years. Time to read the previous month’s *Aesthetic Surgery Journal* stacked in the corner. Time to pick up a hobby or read a novel. Time. All these things I would not have done without this slowdown. Since my daughter was born, I have always wondered what it would be like to be a stay at home mom, especially when I am 20 hours into my day and missed putting her to bed for the third night in a row. That is one gift that this pandemic has given me, a chance to know what the other (supposedly greener) side is. So, while not the pivotal end to my surgical training I expected, an education none the less.

What I have learned is I miss operating…how we used to without N-95 masks. I didn’t expect the last time I would scrub in with my mentors to be filled with fear of operating on an asymptomatic COVID patient. But, I am learning how to let go of the things I cannot control. I think the pandemic today is a lesson in loosening the need for control in everyone. As surgeons, the desire to regulate every aspect of our world can be overwhelming but this pandemic has not shown a possibility. What can be controlled is our response. So, for me, I am choosing to appreciate this gift of time.

With time also comes reflection and the ability to consciously deliberate on the type of plastic surgeon I want to become. In an editorial entitled, “Practice Integrity in a Challenging Economy” by Dr. Nahai written about the financial crisis of 2008, Dr. Nahai states, “some extra soul-searching is definitely in order during an economic slow-down (or any other time) if we catch ourselves doing any of the following,” followed by a list of things, such as operating on patients with unrealistic expectations, risk factors, emotional instability, inappropriate motivations.^2^ As a young surgeon starting out, this is a very important and relevant advice. Especially in times of economic downturn, the desire to get busy and build a practice could, in fact, have the opposite deleterious effect of making poor decisions. Anxiety over not having *any* patients could portend poor patient selection.

So, while today is not the ideal moment to be graduating, perhaps the ideal time does not exist. In a survey-based assessment of the graduating trainees in 2009, Imahara et al^3^ reported that residents’ concern over economic climate did not impact their future career plans despite the turbulent economy. Parallels may be drawn to how graduating residents consider their chosen career paths today. As chief residents, we are fortunate not to be responsible for the livelihood of an entire practice filled with people that have families. We have our entire careers to become busy, prosperous surgeons. The majority of us have plans that include salaried positions. The majority of us are healthy. Perhaps this is the best time to learn the lesson of frugality, necessity, and humility.

When the economy and market do return, there is no doubt that the desire for well-trained plastic surgeons will return. For today, I am healthy and so is my family. Many cannot say the same. Today, I am one step closer to finishing residency, even if it’s not the grass I was expecting to be standing. I hope what all graduating residents can take from this is, at the end of the day we are fortunate. Fortunate to have the opportunities we have and the future ahead of us. As I am writing this, my daughter just threw our $200 baby monitor in the water. A more seasoned stay at home mom would have caught this transgression, so, for now, I will be grateful for the time with her and look forward to the days of missing the grass on this side of the bridge.

## Disclosures

The author declared no potential conflicts of interest with respect to the research, authorship, and publication of this article.

## Funding

The author received no financial support for the research, authorship, and publication of this article.
